# Application of oncoproteomics to aberrant signalling networks in changing the treatment paradigm in acute lymphoblastic leukaemia

**DOI:** 10.1111/jcmm.12507

**Published:** 2014-12-23

**Authors:** Elena López Villar, Xiangdong Wang, Luis Madero, William C Cho

**Affiliations:** a Department of Oncohematology and Pediatrics, Hospital Infantil Universitario Niño Jesús, Universidad Autónoma de MadridMadrid, Spain; b Biomedical Research Centre, Fudan University Zhongshan HospitalShanghai, China; c Department of Respiratory Medicine, Zhongshan Hospital Fudan University School of Medicine, Shanghai Respiratory Research InstituteShanghai, China; d Department of Clinical Oncology, Queen Elizabeth HospitalHong Kong

**Keywords:** acute lymphoblastic leukaemia, personalized medicine, shotgun proteomics

## Abstract

Oncoproteomics is an important innovation in the early diagnosis, management and development of personalized treatment of acute lymphoblastic leukaemia (ALL). As inherent factors are not completely known – *e.g*. age or family history, radiation exposure, benzene chemical exposure, certain viral exposures such as infection with the human T-cell lymphoma/leukaemia virus-1, as well as some inherited syndromes may raise the risk of ALL – each ALL patient may modify the susceptibility of therapy. Indeed, we consider these unknown inherent factors could be explained *via* coupling cytogenetics plus proteomics, especially when proteins are the ones which play function within cells. Innovative proteomics to ALL therapy may help to understand the mechanism of drug resistance and toxicities, which in turn will provide some leads to improve ALL management. Most important of these are shotgun proteomic strategies to unravel ALL aberrant signalling networks. Some shotgun proteomic innovations and bioinformatic tools for ALL therapies will be discussed. As network proteins are distinctive characteristics for ALL patients, unrevealed by cytogenetics, those network proteins are currently an important source of novel therapeutic targets that emerge from shotgun proteomics. Indeed, ALL evolution can be studied for each individual patient *via* oncoproteomics.

## Introduction

Early accurate diagnosis and personalized treatment are essential to treat complex and fatal diseases such as cancer. New paradigms are emerging for innovations on therapies, especially for chronic and malignant diseases such as acute lymphoblastic leukaemia (ALL) *via* shotgun proteomics [1].

ALL can be classified *via* cytogenetic and molecular subgroups for diagnosis, prognosis, and more importantly, for the application of the correct treatment. A remarkable but common in patients with ALL is the appearance of resistance against drugs/therapies. Mutations, genetic aberrations (Table 1) [2–5], cellular heterogeneity of ALL after initial response to therapy and deregulation of signalling networks (unknown for ALL patients with poor responses) play important roles in ALL resistance therapies [6].

**Table 1:** Common acute lymphoblastic leukaemia translocations and cytogenetic abnormalities (http://emedicine.medscape.com/article/207631-workup#a0756) [2–5]

Mutation	Related genes	Survival after 2–3 years (%)
t(10,14)(q24;q11)	HOX11/TCRA	75
6q	Unknown	47
14q11	TCRA/TCRD	42
11q23	MLL	18–26
9p	Unknown	22
12	TEL	20
t(1;19)(q23;p13)	PBX1/E2A	20
t(8;14)(q24;q32)	c-myc/IGH	17
t(2;8)(p12;q24)	IGK/c-myc	80
t(8;22)(q24;q11)	c-myc/IGL	5–10
t(9;22)(q34;q11)	bcr-abl	66
t(4;11)(q21;q23)	AF4-MLL	0–10

Aberrant proteins and signalling networks of ALL evolution can be addressed directly from human body fluids *via* state of art shotgun oncoproteomics. This makes it possible to get the molecules which are not working well or are mutating (space, time) during ALL treatments of each patient. This allows detection of the molecular differences between patients with good response to ALL therapies and patients with poor response. ‘Omics’ shotgun oncoproteomic technologies coupled to cytogenetics are used today in integrated approaches to clarify ALL for future therapy innovations which will benefit ALL patients [7].

For integrating hundreds or thousands of proteomic signatures from ALL patients’ evolution, bioinformatic tools offer different software to create an ALL ‘library’ to link biological discoveries for new drug designs [8].

## ALL treatments tend to be personalized *via* proteomics innovations

With the completion of the human genome project, standard ALL treatments may be adjusted by genetic mutations, including single nucleotide polymorphisms in predicting patient responses (Table 1). In broad sense, individualized medicine is not new, but the options and perspectives have been widely expanded within the last decade *via* omics innovations, including shotgun proteomics, especially when genetic profiles of each ALL patient may modify the susceptibility of therapy [9].

Generally, ALL antimetabolites induce cell death during the S phase of cell growth when incorporated into RNA and DNA or inhibit enzymes needed for nucleic acid production. The drawback involved is that currently, despite the tremendous advances in ALL treatments, patients still suffer from different degree of side effects, toxicities and resistances of treatments [10].

Risk-based ALL therapy (chemotherapy) is emphasized in therapeutic protocols to reduce the toxicity of treatments in patients with good prognosis and provide aggressive therapies for those with poor prognosis. Taken into consideration in the risk stratification include age, initial white blood cells, ALL subtype, chromosomal aberrations or minimal residual disease, although the exact signatures for relapses remain unknown. Chemotherapy and bone marrow transplant are included in the treatment of relapse dependant on those prognostic factors for patients with high risk of early and late relapses with low chemotherapy response. Improved ALL cure rates during the last decades suggest that many relapses in the past were insufficiently treated, in part because of variations in drug disposition, rather than being a reflection of treatment resistance [11].

Central nervous system-directed treatment, and antimetabolite-based maintenance therapy with 6-mercaptopurine (6-MP) and methotrexate, which are continued for up to 2–3 years after diagnosis, is believed to be of major importance for the improved rates of ALL cure [12].

The cytotoxicity of methotrexate relies on cellular depletion of tetrahydrofolates leading to inhibition of nucleotide *de novo* synthesis and amino acid metabolism. Upon intake, 6-MP may become inactivated through methylation by thiopurine methyltransferase. Some methylated 6-MP metabolites (*e.g*. 6-methylthioinosine-monophsophate) also inhibit nucleotide *de novo* synthesis. However, the main cytotoxic effect relies on the purine salvage pathway and kinase-mediated multi-step conversion of 6-MP into 6-thioguanosine nucleotides, which are subsequently incorporated into DNA. Cellular recognition of the resulting nucleobase mismatches induces apoptosis. Thus, innovations in ALL drugs may be achieved *via* shotgun oncoproteomics when mapping the aberrant signalling networks in ALL [13].

Regardless of statistical significance and degree of mechanistic understanding of genotype–phenotype associations, their clinical applicability should be tested in prospective randomized clinical trials. For example, the profile of toxicities in childhood ALL therapy is broad and a limited number of patients are available, it is unrealistic to perform randomized clinical trials for each toxicity, and it is, furthermore, difficult to obtain sufficient statistical power to demonstrate changes in the frequency of rare toxicities. The division of patients into multiple risk groups and the late occurrence of many events further burden such trials. Nevertheless, addressing multiple toxicities and allowing genotype and omics-proteomics-based adjustments of several anticancer agents to reduce the burden of therapy and simultaneously improve cure rates, may be a proof-of-concept approach even though the subsequent statistical and pharmaceutical, plus biological identification, of the most important treatment modifications is a difficult but possible challenge [14].

## Proteomics-based studies for the improvements of ALL therapy

Accordi *et al*. [11] applied reverse phase protein microarrays to identify active-mutated proteins in 118 paediatric B-cell precursor (BCP)-ALL patients. In this study, 92 key signalling proteins have been shown to be activated for this pathology *via* phosphoproteomics. The involved pathways are related to cell proliferation in patients with poor prognosis. BCL-2 is hyperphosphorylated *via* AMPK activation in MLL-rearranged patients. AMPK could have an important role for inhibition of apoptosis in these patients. This is an important issue for innovations in ALL therapy. In addition, Accordi *et al*. [11] realized that prednisone is up-modulating the LCK proto-oncogene, Src family tyrosine kinase (LCK) activity in ALL patients with poor response to this drug. LCK can also play important roles for developing new therapies for ALL patients resistant to prednisone. In addition, Cyclin E is highly expressed in patients suffering relapses at early-phases of the therapy. Thus, there is an important relationship between high level of Cyclin E and relapse incidence. This issue can be complemented *via* shotgun oncoproteomics with future research as it remains critical for improving therapy for ALL patients with relapses [15].

Spleen tyrosine kinase (SYK) is a key molecule controlling apoptosis related to the activation of PI3-K/AKT, NFκB and STAT3 anti-apoptotic signalling pathways in leukaemia type B. Uckun and Qazi [16] carried out a research study where they proposed that SYK might overcome the resistance of malignant B-lineage lymphoid cells to apoptosis providing the theory for more effective multi-modality treatment-therapy regimens for poor prognosis B-precursor ALL (BPL). Radiation by ionizing and different types of chemotherapeutic drugs used in BPL therapy produces DSB in nuclear DNA triggering apoptotic cell death. NFκB and PI3-K survival signalling pathways are activated by chemotherapeutic agents and contribute to drug resistance of leukaemic cells. In addition, NFκB and PI3-K signalling pathways are regulated by tyrosine kinase SYK. SYK phosphorylates SLP-65/BLNK (B-cell linker) are an integral part of effective pre-breakingpoint cluster region (BCR) signalling in BCPs as well as BCR signalling in mature B lymphocytes. SYK plays a relevant regulatory function in early specification and maturation events during B-cell ontogeny. Inhibition of SYK blocks BCR and mTOR signalling pathways, leading to apoptotic death of leukaemic cells. In addition, SYK has another anti-apoptotic role related to leukaemic precursor B cells at the early stages of B-cell (human) ontogeny. Inhibition of SYK triggers apoptosis in primary leukaemic cells from BPL patients who are also resistant to therapy. They carried out studies using a liposomal nanoparticle formulation of a SYK substrate-binding site inhibitor called C61. C61 is coming from ‘nanomedicine’ potential candidate for poor prognosis cases and for relapses BPL cases. C61 was successfully tested in mice as it was able to induce apoptosis in radiation-resistant primary leukaemic cells coming from BPL patients. Uckun and Qazi [16] propose that C61 may be a useful strategy for innovations against ALL therapy refractory. When combining this important current ALL research study to proteomic strategies, signalling networks involved in ALL progression and affected by C61, could be discovered; thus, key signalling molecules ALL can be identified for good new specific target candidates [16].

When Notch signalling is abnormally activated/deactivated, this implies a relevant oncogenic mechanism for ALL T cells. ALL subtype T is commonly quite aggressive, especially for disease in children. Lin and coworkers [13] *via* proteomic assays identified DDX5, an ATP-dependent DEAD-box RNA helicase, and MAML1 protein. DDX5 has been shown to be associated with the endogenous NOTCH1 transcription activation complex in human T-ALL leukaemic cells. Thus, unravelling the molecular regulation of Notch signalling is crucial to identify new approaches to block aberrant Notch oncogenic activity during ALL progression. MAML transcriptional activator is critical for signalling activation of Notch. Indeed, MAML1 is the one involved in the regulation of Notch in leukaemic cells although its mechanism remains unknown. Lin *et al*. [17] also proved that lentivirus-mediated short-hairpin RNA knock-down of DDX5 was because of low expression of Notch target genes, decreased cell proliferation and increased apoptosis in cultured human leukaemic cells together with signalling of Notch activation. This study demonstrates that DDX5 is pushing for useful Notch-mediated transcription in leukaemic cells. Therefore, DDX5 can be a future potential new target therapy for regulating Notch signalling in leukaemia [17].

PI3K/AKT pathway mutations have been found in T-cell ALL. Nevertheless, their relevance related to other genetic aberrations is not yet clear. *PTEN* mutations are proposed as secondary mutations which follow NOTCH1-activating mutations and later on, they can produce cellular resistance to γ-secretase inhibitors. Zuurbier *et al*. [18] investigated the role of *PTEN*, *PI3K* and *AKT* aberrations in paediatric T-cell leukaemia patient cohort (*n* = 146) treated on DCOG or COALL protocols. The authors discovered that *PTEN* and AKT E17K aberrations appeared in around 13% and 2% of patients respectively. They realized that *PTEN/AKT* mutations appeared in a high percentage in *TAL*- or *LMO*-rearranged leukaemia, although *PTEN/AKT* mutations did not appear in *TLX3*-rearranged patients (*P* = 0.03). The opposite data resulting was obtained for NOTCH1-activating mutations. In addition, Zuurbier *et al*. [14] detected that T-cell leukaemia patients without *PTEN/AKT* and NOTCH1-activating mutations fared well, with a cumulative incidence of relapse of only 8% *versus* 35% for PTEN/AKT and/or NOTCH1-activated patients (*P* = 0.005). This is critical and important information related to the significance of *PTEN* and *AKT* aberrations in paediatric T-cell ALL. Applying quantitative phosphoproteomics strategies to peripheral blood and bone marrow ALL, several signalling pathways (and networks) can be captured in a single experiment. Therefore, hundreds of phosphorylated protein kinases can be detected if they are working well during ALL or we can even get those which do not operate properly from each ALL patient evolution. This is key information for patients suffering relapses and resistance or toxicity to the current ALL treatments [18].

Braoudaki *et al*. [19] developed proteomic studies to see the differential proteins expressed when comparing low- and high-risk patients suffering ALL. Cytogenetic assays were carried out in parallel to proteomics to get complementary data for clinical advances. Proteins were extracted from bone marrow and peripheral blood plasma of patients who belong to high- and low-risk ALL at diagnosis. They applied 2DE (2 Dimentional Electrophoresis) coupled to MALDI-MS/MS analysis and, later on, the differentially expressed proteins detected were validated *via* Western blot. Proteins Clus, Ceru, ApoE, ApoA4, ApoA1, Gels, S10A9, Ambp, Actb, Cata and Afam have an important role in leukaemia prognosis, mainly as distinctive signals for aggressive leukaemia cases. None of these identified biomarkers for ALL, as distinctive signals for aggressive cases, are currently evaluated routinely at hospitals. It could be interesting to check the previously resulting data of Braoudaki *et al*. [15] for each patient when he/she is diagnosed with ALL. If more patients could be checked for these biomarkers, more statistical and clinical value would be achieved, thus, ensuring true biomarkers useful for therapy improvements. We would change 2DE for ESI-LC-MS as it is faster and with higher accuracy despite the important ALL information provided [19].

Peripheral blood plasma was shown to be a good sample to predict clinical behaviour in ALL patients irrespective of the percentage of bone marrow blasts. Albitar *et al*. [20] analysed, *via* proteomics, this clinic sample type from 57 patients suffering ALL before initiation of therapy. They applied strong anion exchange coupled to protein chip arrays and surface-enhanced laser desorption/ionization. It was shown that recurrence prediction is independent to bone marrow blast count, cytogenetic assays and surface markers. Thus, evolution ALL responses of patients can be followed *via* proteomic tools to cover gaps which nowadays are not explained by genotype–phenotype assays (Table 1).

Also, proteomic research in paediatrics is important and most of the successes thus far are seen in research that utilize samples that require less invasive procedures and focus on prevailing childhood diseases such as ALL. Nevertheless, most of platelet proteome data obtained to date are derived from the adult population and the potential of platelet proteomic application in children has not yet been explored [21].

## Future perspectives

Signalling networks do not operate in an independent way, as signalling cascades are connected. Thus, we defined a space (bone marrows and peripheral blood) and time-based strategies (at several states of the patients) to understand the ALL evolution of each patient and to capture the dynamics of phosphorylation events, from which useful targets can be discovered for treating ALL.

A shotgun oncoproteomic strategy, space and time-based, using sequential elution from immobilized metal affinity chromatography (SIMAC) [22] and isobaric tag for relative and absolute quantitation (iTRAQ) [23] coupled to liquid chromatography, electrospray ionization, tandem mass spectrometry (LC-ESI-MS/MS) [24,25] is proposed to be applied in our research team by the Spanish Health System (SNS) in collaboration with international research teams to unravel signatures of ALL patients who do not respond well to treatments compared to those who do.

SIMAC coupled to iTRAQ and LC-ESI-MS/MS will allow us to identify the up- and down-regulated phosphorylated proteins *via* highly sensitive techniques. The resulting data should show us important clinical and biological information of signalling networks involved in ALL resistances and toxicities.

**Fig. 1:**
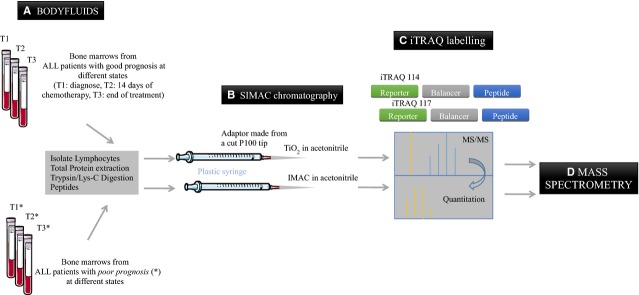
Flow-through routine analysis of blood for leukaemia diagnose *via* oncoproteomics. Blood samples from acute lymphoblastic leukaemia (ALL) patients are routinely analysed by microscopy, cytometry, FISH, karyotype and polymerase chain reaction. These assays are well-established to detect leukaemic cells, specific translocations and rearrangements. The ALL treatment is adequate according to the genetic data (with high or low chemotherapy dose according to the high, intermediate and low risk of relapse). When combining those previously routine assays to shotgun proteomic analyses for each patient, coupling SIMAC, iTRAQ and ESI-LC-MS/MS, real protein biomarkers of ALL evolution per each patient can be discovered. Thus, new therapy innovations may appear.

**Fig. 2:**
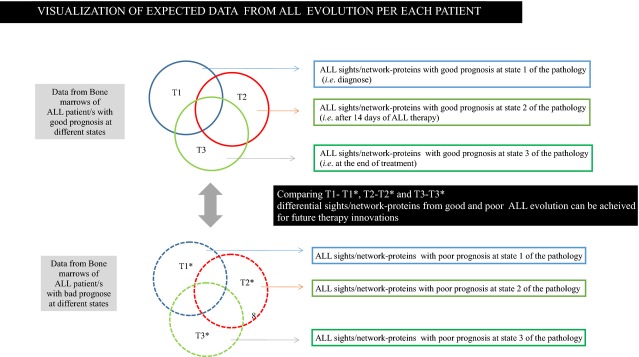
Putative visualization of oncoproteomics ALL resulting data to several patients at different acute lymphoblastic leukaemia (ALL) states (diagnose, 14 days after treatment and at the end of the treatment). Comparing different ALL states per each patient, ALL signatures evolution can be achieved, thus, unknown ALL molecules which are not working properly can be detected. In addition, when comparing the resulting data from ALL patients with good and poor prognosis, distinctive signatures involved in ALL progression can be identified. Therefore, therapy innovations can be achieved.

We expect that this research strategy will help to improve current ALL treatments, especially for ALL patients with poor prognoses (Figs 1 and 2). Flow-through routine analysis of blood for leukaemia diagnose *via* oncoproteomics and putative visualization of oncoproteomics ALL resulting data to several patients at different ALL states (diagnose, 14 days after treatment and at the end of the treatment) are detailed in both figures. Comparing different ALL states per each patient, ALL signatures evolution can be achieved, thus, unknown ALL molecules which are not working properly can be detected. In fact, when comparing the resulting data from ALL patients with good and poor prognosis, distinctive signatures involved in ALL progression can be identified. Therefore, therapy innovations can be achieved. As delegate of HUPO, for human proteome on children ALL studies at Hospital Universitario Niño Jesús, we are pursuing to support the human proteome in this context. We envision this will further benefit the understanding of the pathology of the disease and ultimately improve the diagnoses and personalized treatment.

Selected/multiple reaction monitoring [26–28], Western blotting and ELISA assays can also help to validate identified signatures for each ALL patient from shotgun oncoproteomic strategies.

The resulting data per patient can be correlated and linked to other patients. Thus, a ‘library’ collection of ALL evolution for patients can be set-up, which will help to ultimate a reference, once a statistically significant number of ALL patients is studied.

**Fig. 3:**
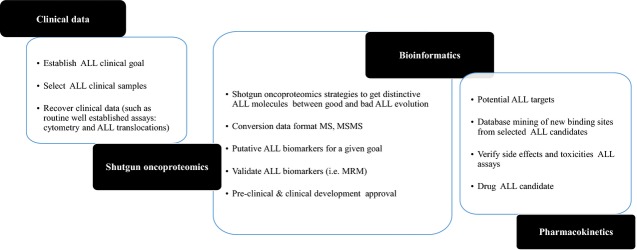
Schedule from clinical to oncoproteomics and bioinformatics analyses. Once the clinical goal and human body fluids have been selected from ALL patients, efficient shotgun oncoproteomics must be established and be coupled to bioinformatic tools, which can be applied for the potential development of personalized approaches, aimed at the concept of personalized ALL medicine. After long and tedious assays, new ALL targets can be discovered, and database mining from new binding sites from ALL candidates can be created. Also, verification of side effects and toxicities for the new ALL molecules have to be studied, to validate a new ALL drug candidate.

Once the phosphorylation state of network proteins, constitutive or associated to ALL evolution is established by shotgun oncoproteomics, a range of bioinformatic methods permits deeper study of its properties and contacts. Using sequence analysis, sequence comparison, virtual approaches of protein–protein, protein–ligand interaction or molecular dynamics simulations, initial physical information can be applied for the potential development of personalized approaches, aimed at the concept of personalized ALL medicine. Bioinformatics covers a wide spectrum of techniques for the generation and use of beneficial information from structure, sequence or relationships among biological items (DNA, RNA, proteins, macromolecular complexes, *etc*.) [29,30]. Those most useful in clinical ALL studies are: Ascore and PhosphoScore (statistical algorithms which measure the probability of correct phosphorylation site localization based on the presence and intensity of site-determining ions in MS2 spectra), and next-generation sequencing (NGS) was used in a detailed study of genes involved in colorectal cancer [31]. As a main conclusion of the study, the authors stated that sequencing of whole tumour exomes allowed prediction of the microsatellite status of trinucleotide CGC, facilitating, at the same time, the putative finding of relevant mutations. NGS can be applied to formalin-fixed and paraffin-embedded material, allowing the renewed study of all the ancient material stored in the pathology departments), sequence-to-sequence and sequence-to-structure comparisons (multiple sequence analysis) to obtain valuable information on the nature of the functional implications of the mutated residues in the protein ALL context. Homology modelling 3D structure of the active centre of a protein of interest (in absence of experimental crystal structures, the homology modelling methods), can develop a 3D model from a protein sequence based on the structures of a crystallized homologous protein. Information on the 3D structure of the active centre of a protein of interest and/or its natural ligands can be used as a basis for the design of effective drugs and the more sophisticated rational drug design and molecular dynamics techniques. Using shotgun oncoproteomics together with structural analysis of proteins and bioinformatic tools, important biological understanding of ALL evolution can be achieved. Prototypical shotgun oncoproteomic coupled to bioinformatics pipeline useful for clinical ALL research is illustrated [32,33] (Fig. 3). In the schedule of Figure 3, it is illustrated in a simple manner that firstly, the clinical goal and human samples have to be established and selected from ALL patients; secondly, an efficient shotgun oncoproteomics must be designed and coupled to bioinformatic tools to get biomarkers for a given clinical issue, and to arrange the resulting data. From the resulting data, potential ALL new targets can be achieved, and therefore new ALL drug candidates can be designed and validated. In fact, verification of side effects and toxicities for the new ALL molecules have to be studied, to validate a new ALL drug candidate, aimed at the concept of personalized ALL medicine.

## Conclusions

Network proteins with distinctive characteristics for ALL patients, unrevealed through cytogenetics, are currently an important source of novel therapeutic targets that emerge from shotgun proteomics.

Shotgun oncoproteomics and bioinformatic strategies need to be combined to achieve the personalized medicine. Studies of reversible phosphorylation in proteins from ALL networks will allow the generation of models for protein–protein contacts at the molecular level taking into account each particular protein sequence. Molecular dynamic analysis of those contacts will allow the modification of the 3D computer models obtaining virtual structures tailored to individual patients. So, we should get, according to the resulting structure information, new drug candidates for adapting the therapies; and innovate new ALL treatments. In fact, the ALL evolution per each patient can be studied *via* shotgun oncoproteomics, thus ALL patients can be benefited.
